# Intermittent hypoxia-induced enhancement of sociability and working memory associates with CNTNAP2 upregulation

**DOI:** 10.3389/fnmol.2023.1155047

**Published:** 2023-04-06

**Authors:** Qing Zhang, Lu Xu, Yang Bai, Peiye Chen, Mengen Xing, Fang Cai, Yili Wu, Weihong Song

**Affiliations:** ^1^Oujiang Laboratory (Zhejiang Lab for Regenerative Medicine, Vision and Brain Health), Institute of Aging, Key Laboratory of Alzheimer’s Disease of Zhejiang Province, Zhejiang Provincial Clinical Research Center for Mental Disorders, School of Mental Health and Kangning Hospital, Wenzhou Medical University, Wenzhou, Zhejiang, China; ^2^Townsend Family Laboratories, Department of Psychiatry, Brain Research Center, The University of British Columbia, Vancouver, BC, Canada

**Keywords:** CNTNAP2, hypoxia, transcription, sociability, memory

## Abstract

**Introduction:**

Hypoxia is an environmental risk factor for many disorders throughout life. Perinatal hypoxia contributes to autism spectrum disorder (ASD), while hypoxic conditions in the elderly facilitate memory deficits. However, the effects of hypoxia on adolescence remains elusive. CNTNAP2 is a critical molecule in ASD pathogenesis with undefined mechanisms. We investigate hypoxia’s impact on adolescence and the underlying mechanism related to CNTNAP2.

**Methods:**

Three-chamber social approach test, Y maze, Morris Water Maze and Open Field Test were applied to evaluate behavioral alterations. Immunoblotting, 5′- RACE and dual-luciferase reporter assay were performed to examine CNTNAP2 protein expression, transcription start site (TSS) of human CNTNAP2 gene and CNTNAP2 promoter activity, respectively.

**Results:**

Intermittent hypoxia treatment improved social behaviors and working memory in adolescent mice. CNTNAP2 was increased in the brains of hypoxia-treated mice. The sequencing results identified the TSS at 518 bp upstream of the translation start site ATG. Hypoxia upregulated CNTNAP2 by interacting with functional hypoxia response elements in CNTNAP2 promoter.

**Conclusion:**

Intermittent hypoxia enhanced sociability and working memory associated with CNTNAP2 upregulation. Our study provides novel insights into intermittent hypoxia’s impact on development and the interaction between genetic and environmental risk factors in ASD pathogenesis.

## Introduction

Oxygen homeostasis is crucial for the survival of organisms, and the brain is especially susceptible to hypoxia due to its vast demand for oxygen (Sharp and Bernaudin, 2004). Hypoxia occurs in many pathophysiological conditions in the brain throughout the lifetime, such as perinatal hypoxia-ischemia encephalopathy, sleep apnea, and stroke. The impact of hypoxia on the brain’s development and function is also manifested in an age-dependent manner. Abundant evidence has shown that hypoxia exposure causes neurodevelopmental deficits in rats (Moromisato et al., 1996; Wilson et al., 2022) and infants (Wang et al., 2018; Dassios et al., 2022; Plomgaard et al., 2022) at prenatal and perinatal stages, contributes to brain myelin injury in adult mice (Chen et al., 2021) and brain white matter injury in adult human (Muttikkal and Wintermark, 2013), and exacerbates memory impairment in aging mice (Sun et al., 2006) and human (Park et al., 2013). However, few studies have addressed the impact of hypoxia on adolescents, especially brain functions during this developmental stage. Current understandings of hypoxia’s impact on adolescents mainly focus on disease models. For example, hypoxia facilitates impairments in adolescents who have suffered from underlying diseases, such as sickle cell disease (Halphen et al., 2014) and cystic fibrosis (Urquhart et al., 2005). Hypoxia’s impact on healthy adolescence and the possible underlying mechanism remains unknown. Therefore, this study investigated hypoxia’s effect on healthy adolescent mice and potential molecular mechanisms.

Although the exact mechanisms underlying hypoxia’s impact on brain functions remain unclear, transcription factor hypoxia-inducible factor 1 (HIF-1) is the unquestionable regulator of the brain’s response to hypoxia, like elsewhere in the body (Bonkowsky and Son, 2018). HIF-1 is a helix-loop-helix transcription factor with a constitutively expressed HIF-1β subunit and a hypoxia-induced HIF-1α subunit (Wang et al., 1995). Under normoxic conditions, HIF-1α is continuously produced and quickly degraded (half-life about 5 min) by the ubiquitin-proteasome pathway (Huang et al., 1998) via HIF prolyl-hydroxylases (HPHs). Hypoxia inhibits HPHs, thereby stabilizing and accumulating HIF-1α (Bruick and McKnight, 2001; Epstein et al., 2001). HIF-1α is then phosphorylated and binds to HIF-1β. Next, the HIF-1α/HIF-1β dimer forms a complex with p300/CBP, and the complex binds to the hypoxia response element (HRE) with a core DNA sequence 5′-A/(G)CGTG in the promoters of target genes to activate their transcriptions (Wang et al., 1995; Sharp and Bernaudin, 2004). Transcriptional regulation by HIF-1α is the essential mechanism underlying hypoxia’s effects in physiological and pathological conditions. For example, erythropoietin (EPO) is upregulated by HIF-1α to boost erythropoiesis under anemic conditions or at high altitudes (Wang et al., 1995), while hypoxia exacerbates memory deficits in Alzheimer’s Disease (AD) mouse model by upregulating BACE1 (Sun et al., 2006).

As an environmental risk factor for neurodevelopmental disease, hypoxia has been intensively studied in prenatal and perinatal stages. Autism spectrum disorder (ASD) is a group of neurodevelopmental disorders characterizing social communication deficits and repetitive/restrictive behaviors and interests (Lord et al., 2020). In clinical studies, pregnancy and birth complications associated with hypoxic-ischemic damage are strongly related to ASD development (Kolevzon et al., 2007; Modabbernia et al., 2017). Of note, respiratory distress and other hypoxia markers in prenatal and perinatal stages are strongly associated with increased ASD risk in twins, whose shared risk factors such as parents’ age, medication, and pregnancy complications were well-matched (Froehlich-Santino et al., 2014).

*CNTNAP2* gene locates in chromosome 7, encoding an eponymous protein belonging to the neurexin superfamily. CNTNAP2 protein was initially found as an adhesion molecule connecting neurons and glial cells (Poliak et al., 1999). Recent studies broadened the knowledge on CNTNAP2 to a novel synaptic protein with multiple functions. For example, CNTNAP2 is required for axonal growth (Canali et al., 2018), and it stabilizes dendrites in interneurons via interacting with CASK (Gao et al., 2018). *CNTNAP2* is one of the most referred genes in ASD, with increasing variations identified in ASD patients (Bakkaloglu et al., 2008; Penagarikano and Geschwind, 2012). *Cntnap2* knockout mice and rats have aberrant communicative, social, and repetitive behaviors, strikingly mimicking ASD in humans (Penagarikano et al., 2011; Scott et al., 2020). Additionally, reduced hippocampal PV interneuron density and inhibitory input to CA1 pyramidal cells were noticed in *Cntnap2* knockout mice (Paterno et al., 2021). The expression of CNTNAP2 is significantly decreased in ASD patients (Sampath et al., 2013; Maekawa et al., 2015). Additionally, variants in the *CNTNAP2* promoter region were identified in ASD patients, which presumably caused the altered CNTNAP2 expression levels (Nord et al., 2011; Chiocchetti et al., 2015). However, the transcriptional regulation of CNTNAP2 expression remains largely unknown.

Adolescence is a critical developmental stage, both physically and mentally. During this second ‘window of opportunity’ in brain development, memory and social behaviors can be sensitively affected by environmental factors (Fuhrmann et al., 2015), while the related knowledge is sparse. In this study, we investigated hypoxia’s impact on social behaviors and memory in adolescent mice and potential mechanisms. First, we observed that intermittent hypoxia treatment enhanced social behaviors and working memory in adolescent mice. Next, we found that CNTNAP2 was increased in the mouse brain. Finally, we demonstrated that hypoxia upregulated CNTNAP2 expression by interacting with the functional HREs in the *CNTNAP2* promoter.

## Materials and methods

### Plasmid constructs

A 2,868 bp (−2,431 bp to +437 bp) 5′-flanking region of the human *CNTNAP2* gene, named pCNTNAP2-A, was amplified from the Human Embryonic Kidney 293 (HEK) cell genomic DNA by PCR and cloned into the pGL3-Basic vector (Promega Cat# E1751). A series of deletion fragments were sub-cloned into the pGL3-Basic vector upstream of the luciferase reporter gene using pCNTNAP2-A as the template and proper restrictive enzymes. The primers with restrictive enzyme sites were synthesized as listed in Table 1. To determine the functional HRE, triple CNTNAP2-HRE1 to HRE5 with flanking regions (18 bp) were cloned into the pGL-pL vector generated in our previous publication (Cai et al., 2008) by annealing two reverse-compliment primers (Table 2). pHA-HIF-1α plasmid expresses HA-tagged HIF-1α (Huang et al., 1998).

**TABLE 1:** Primers for cloning human *CNTNAP2* promoter constructs in the pGL3-Basic vector.

Name	Sequence 5′–3′	Construct
-2431MluI-F	cgacgcgtccagttttgctggaccgtgt	pCNTNAP2-A
-2046MluI-F	cgacgcgtatatggcacagaggtggtgc	pCNTNAP2-B
-1460MluI-F	cgacgcgtgaggcagttcacctcccatt	pCNTNAP2-C
-868KpnI-F	gcctcaggtaccttgaaatagttacc	pCNTNAP2-D
-298MluI-F	cgacgcgtagggcagaagggttttgaca	pCNTNAP2-E, F
-198MluI-F	cgacgcgtaagagaagggagggcttcc	pCNTNAP2-G, H
-6MluI-F	cgacgcgtccacatacacaagctctcc	pCNTNAP2-I
+437BglII-R	gaagatcttgggctgtcctcaaatacgc	(Reverse primer)
+51XhoI-R	gtgactcgaggggtttccact	(Reverse primer)

**TABLE 2:** Primers for annealing CNTNAP2-HRE.

Name	Sequence 5′–3′
HRE1-*Nhe*I-F	ctagctgtatgcgtgtttgcctatgtatgcgtgtttgcctatgtatgcgtgtttgcctac
HRE1-*Xho*I-R	tcgagtaggcaaacacgcatacataggcaaacacgcatacataggcaaacacgcatacag
HRE2-*Nhe*I-F	ctagccagcagcgtgtgtttggtcagcagcgtgtgtttggtcagcagcgtgtgtttggtc
HRE2-*Xho*I-R	tcgagaccaaacacacgctgctgaccaaacacacgctgctgaccaaacacacgctgctgg
HRE3-*Nhe*I-F	ctagcggtgtacgtgtgcatatgggtgtacgtgtgcatatgggtgtacgtgtgcatatgc
HRE3-*Xho*I-R	tcgagcatatgcacacgtacacccatatgcacacgtacacccatatgcacacgtacaccg
HRE4-*Nhe*I-F	ctagcgccgtgcgtgcgccccgggccgtgcgtgcgccccgggccgtgcgtgcgccccggc
HRE4-*Xho*I-R	tcgagccggggcgcacgcacggcccggggcgcacgcacggcccggggcgcacgcacggcg
HRE5-*Nhe*I-F	ctagccctcgagtcacgctgccccctcgagtcacgctgccccctcgagtcacgctgcccc
HRE5-*Xho*I-R	tcgaggggcagcgtgactcgagggggcagcgtgactcgagggggcagcgtgactcgaggg

### 5′-RACE assay

Total RNA was extracted from a human fetus brain sample by TRI reagent as instructed by the manufacturer (Sigma). 5′-RACE assay was conducted using the Smarter RACE 5′/3′ kit (Clontech) following the user manual. 1 ug total RNA was used to synthesize the 5′-RACE-Ready first-strand cDNA template. Using this cDNA template, 5′ rapid amplification of cDNA ends (RACE) was performed through PCR reactions with universal forward primer (provided in the kit) and the CNTNAP2-specific reverse primer (5′-GATTACGCCAAGCTT aagaggaaggcagagcgacggggacggt). The 5-RACE product was cloned into the pRACE vector via In-Fusion HD Cloning. The transcription start site (TSS) was mapped by sequencing independent colonies from the cloning.

### Cell culture, cell transfection, and hypoxia treatment

Human embryonic kidney 293 (HEK) cell line (RRID:CVCL_0045) was cultured in Dulbecco’s Modified Eagle Medium (Cytiva Cat# SH30243.01) supplemented with 10% fetal bovine serum (Gibco Cat# 12483020), 100 U/mL Penicillin-Streptomycin (Gibco Cat# 15140122). Rat pheochromocytoma-derived PC12 cell line (RRID:CVCL_F659) was maintained in the same DMEM medium supplemented with 15% fetal bovine serum. Cells were seeded in a 48-well plate at the seeding density of 0.05 × 10^6^/well and maintained in an incubator at 37°C with 5% CO_2_. Cell transfections were performed with Polyethylenimine. For hypoxia treatment, transfected cells were maintained at 37°C in an incubator with 21% O_2_ as the control treatment or in an incubator with 2% O_2_ as the hypoxia treatment for 24 h.

### Dual-luciferase reporter assay

To examine the promoter activity for pCNTNAP2 constructs, PC12 cells were co-transfected with 250 ng Firefly luciferase plasmids (pGL3-Basic, pCNTNAP2 plasmids) and 0.5 ng Renilla luciferase plasmid pCMV-Luc to control for the transfection efficiency. To examine the effects of HIF-1α on *CNTNAP2* promoter activity, HEK cells were co-transfected with 250 ng Firefly luciferase plasmids, 250 ng HIF-1α, or empty vectors, and 1 ng Renilla luciferase plasmid. Twenty-four hours after transfection, cells were harvested and lysed in 1× passive lysis buffer (100 μl/well). Firefly and Renilla luciferase activities were sequentially measured using the Dual-Luciferase Reporter Assay System (Promega Cat# E1980). Relative luciferase units (RLU) were calculated by normalizing Firefly luciferase activities to the corresponding Renilla luciferase activities to reflect the relative promoter activities.

### Animals and hypoxia treatment

C57BL/6J mice (4 weeks old, RRID:IMSR_JAX:000664) were group-housed with free access to food and water. Mice were randomly assigned to control and hypoxia treatment groups. The hypoxia treatment was performed in a hypoxia chamber (TOW-INT TECH, Cat# PR00X-100-S) with an 8% O_2_ supply, 16 h/day for 2 weeks (6-week-old upon behavioral tests). Mice were euthanized using isoflurane with care to reduce the suffering. Brains were extracted, snap-frozen and kept in – 80°C for the following experiments. The animal experiments were approved by the University of British Columbia Animal Care and Use Committee (ID: A15-0229) and Animal Experimental Ethical Inspection of Laboratory Animal Center, Wenzhou Medical University (ID: xmsq 2021-0003).

### Immunoblotting

Brain samples were homogenized and lysed in RIPA lysis buffer supplemented with a protease inhibitor cocktail (Roche from Sigma-Aldrich Cat# 11873580001). Protein concentration was measured by protein assay. The lysates were resolved on 8% Tris-glycine SDS-PAGE and transferred onto nitrocellulose membranes. Membranes were blocked in 5% non-fat milk in PBS for 1 h at room temperature, followed by blotting with primary antibodies overnight (16 h) at 4°C. The following day, membranes were washed with washing buffer Tris-buffered saline with 0.1% Tween-20 (TBST), then blotted with secondary antibodies for 1 h at room temperature. After washing with TBST, images were developed using ECL. Primary antibodies: anti-Caspr2 (D6S10) (1: 1,000, Cell Signaling Technology Cat# 61962, RRID:AB_2799616), β-Actin (8H10D10) (1: 1,000, Cell Signaling Technology Cat# 3700, RRID:AB_2242334). Secondary antibodies: goat anti-mouse IgG (ZSGB-Bio Cat# ZB-2305, RRID:AB_2747415), goat anti-rabbit IgG (ZSGB-Bio Cat# ZB-2301, RRID:AB_2747412).

### Three-chamber social approach test

The test was performed as previously described (Kaidanovich-Beilin et al., 2011). The day before experiments, novel mice were trained in cages located in two side chambers. On the testing day, subject mice were first allowed to freely explore the three-chamber apparatus for 10 min to habituate. In stage I sociability test, subject mice were initially placed in the center chamber and then allowed to visit an empty cage and a novel mouse (stranger 1) in two opposite side chambers for 10 min. In stage II social novelty preference test, the empty cage was replaced by another novel mouse (stranger 2), and the subject mice freely moved in the three-chamber apparatus for 10 min. The behaviors were recorded and analyzed using Any-maze (Stoelting).

### Y maze

Y maze was performed to investigate spatial working and reference memory as described (Kraeuter et al., 2019). Mice were placed in the center and allowed to visit three arms freely for 10 min. The behaviors were recorded and analyzed using Any-maze (Stoelting). Three consecutive entries into different arms were considered one successful alteration. The total number of events is the total number of entries into arms minus two. The spontaneous alteration reveals the ratio of mice visiting three different arms in a row (number of successful alteration) in all events.

### Morris Water Maze

Morris Water Maze was used to assess learning and spatial memory. The test was performed in a circular pool (1.2 meters in diameter, 0.5 meter in depth) filled with opaque water as previously described (Bromley-Brits et al., 2011). The platform (9 cm in diameter) was in the southwestern (SW) quadrant of the pool, 1 cm above the water on day 1, 1 cm below the water on days 2-5, and was removed on day 6. In the visible platform test on day 1, mice were trained for 5 trials (60 seconds per trial) to reach the platform. In the hidden platform test on days 2-5, mice were allowed to freely search for the platform for 60 seconds. Each mouse received 5 trials from 4 points of the pool with an interval of 60 min. In the probe test on day 6, mice were allowed to start from the same point and swim freely for 60 seconds to search for the removed platform. The behaviors were recorded and analyzed using Any-maze (Stoelting).

### Open field test

Open filed test was conducted to detect the locomotor activity and anxious behavior change. Mice were put in an open field (50 cm × 50 cm) for 10 min free exploration. The open field is divided into 25 squares of equal area; each 10 cm × 10 cm is a grid. The four squares near the corner are set as corners, the 9 squares in the middle are set as the center area, and the remaining 12 squares are set as the edge area. Any-maze (Stoelting) was used to record the routes, speed, distance, and the time in center zone.

### Statistics

All the data are expressed as mean ± standard error of the mean (SEM). Replicate numbers (*n*) are shown in the figure legends. GraphPad Prism 8 was used for statistical analyses and quantification graphs. One-way or two-way analyses of variance (ANOVA) were used to analyze multiple comparisons, followed by *post-hoc* multiple comparisons tests specified in the figure legends. Sidak’s multiple comparisons test was applied for the analyses of three-chamber approach test, Morris Water Maze test and promoter activity. In three-chamber approach test, the treatment and subject are the independent variable; the interaction time is the dependent variable. In Morris Water Maze test, the treatment and time (Day 2–5) are the independent variable; the latency or distance is the dependent variable. In the promoter activity analysis, two independent variables are the treatment and different fragments, while dependent variable is promoter activity. A *p*-value less than 0.05 was considered statistically significant.

## Results

### Intermittent hypoxia treatment increased sociability and enhanced working memory

**FIGURE 1:**
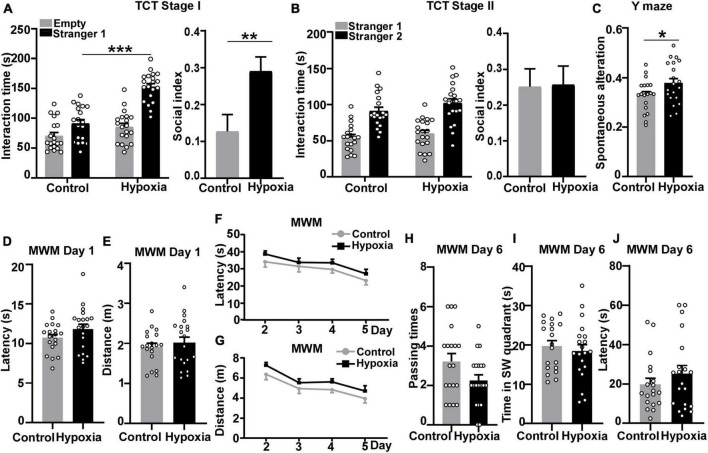
Intermittent hypoxia treatment enhanced sociability and working memory in mice. 1-month-old C57BL/6J mice were treated with 8% O_2_, 16 h/day for 2 weeks, followed by behavior tests at 6 weeks old. Three-chamber social approach test was performed to detect the impact of hypoxia on sociability and social novelty preference. **(A)** Stage I sociability test. Mice were treated with normoxia (*n* = 9 male + 10 female) and hypoxia (*n* = 10 male + 10 female) treatments both spent more time interacting with stranger 1. Two-way ANOVA was applied and treatment is considered as an independent variable, *F* (1, 74) = 40.99, *p* < 0.001. We calculated the ratio of the difference to the sum of time interacting with stranger mouse 1 and empty cage to represent the social index in stage I (unpaired *t*-test). **(B)** Stage II social novelty preference test. Mice under control and hypoxia treatments both spent longer time interacting with stranger 2. Two-way ANOVA was used and treatment effect is an independent variable, *F* (1, 74) = 2.467, *p* = 0.1205. Unpaired *t*-test was applied to calculate the ratio of the difference to the sum of time interacting with stranger mouse 1 and stranger mouse 2 in stage II. **(C)** Y maze was carried out to examine spatial working memory and reference function. Spontaneous alteration represents the ratio of mice entering three different arms in a row. P values were calculated by unpaired *t*-test. **(D–J)** Morris Water Maze test was performed to assess spatial learning and memory. **(D,E)** Day 1 visible platform. Mice were trained to find the visible platform to exclude motor or visual obstacles. No differences between control or hypoxia-treated mice were found in terms of latency **(D)** or distance **(E)**. Unpaired *t*-test was used to calculate *p*-value. **(F,G)** Day 2 to day 5 hidden platform. Mice were trained to find the hidden platform under the non-transparent water surface. After 4-day training, mice traveled shorter distances and latencies to find the platform. Hypoxia treat mice spent similar latency (Two-way ANOVA, treatment effect, F (1, 148) = 4.299, *p* = 0.0399) and distance [Two-way ANOVA, treatment effect, *F* (1, 148) = 6.410, *p* = 0.0124] to reach the platform compared with the control mice. **(H–J)** Day 6 probe test. The hidden platform was removed, and mice were allowed to freely explore for 60 s. No significant differences were found between the two treatment groups in terms of platform passing times **(H)**, time in the SW quadrant **(I)**, and latency of first entry to the platform zone **(J)**. *p*-Values were calculated by unpaired t-test. The values represent means ± SEM. *p < 0.05, **p < 0.01, ***p < 0.001.

To investigate whether hypoxia affects social behavior and memory in adolescent mice, 1-month wildtype C57BL/6J mice were treated with intermittent hypoxia (8% O_2_, 16 h/day) for 14 days, followed by behavior tests at 6 weeks old. First, three-chamber social approach test was performed to examine sociability and novelty preference. In stage I sociability test, both control and hypoxia-treated mice spent more time interacting with stranger 1 than with the empty cage, indicating normal sociability. Two-week hypoxia treatment did not affect the time spent with empty cage, *p* = 0.1530 (Figure 1A). However, hypoxia-treated mice spent significantly more time interacting with stranger 1 than control mice, *p* < 0.001 (Figure 1A). Furthermore, we calculated social index to measure the alteration in sociability. Mice under hypoxia exposure manifested higher social index compared with the control group (control, 0.1267 ± 0.04598; hypoxia, 0.2906 ± 0.03789), *p* = 0.0089 (Figure 1A), suggesting that intermittent hypoxia enhances the sociability of adolescent mice. In stage II social novelty preference test, two groups of mice displayed significant preference to novel mouse stranger 2, indicating normal social novelty preference. Hypoxia-treated mice spent similar time interacting with stranger 1, *p* = 0.7126 (Figure 1B) and stranger 2, *p* = 0.2643 (Figure 1B) as the control mice, and showed comparable social index with the control ones (control, 0.2520 ± 0.04961; hypoxia, 0.2575 ± 0.05179), *p* = 0.9396 (Figure 1B), indicating that hypoxia treatment did not affect social novelty preference in adolescent mice. These results implicate that the 2-week hypoxia treatment increases sociability in adolescent mice. Besides, we conducted the open field test to assess locomotor activity and anxious behavior. Two groups showed no significant differences in the distance, *p* = 0.6077 (Supplementary Figure 1A) and speed, *p* = 0.6155 (Supplementary Figure 1B), indicating that hypoxia did not affect the motor function. Hypoxia-stimulated mice spent comparable time in the center zone as the control mice, supporting the stable emotional behavior, *p* = 0.4164 (Supplementary Figure 1C). Similar outcomes were detected in both sexes. Male mice stimulated by hypoxia resembled the control mice in locomotor activity and emotional changes. Distance, *p* = 0.8541 (Supplementary Figure 1D). Speed, *p* = 0.8089 (Supplementary Figure 1E). Time in the center zone, *p* = 0.0605 (Supplementary Figure 1F). Similarly, female mice treated with hypoxia had comparable performance with the control ones (Supplementary Figure 1G, *p* = 0.5841; Supplementary Figure 1H, *p* = 0.6285; Supplementary Figure 1I, *p* = 0.2462). These results suggest that the 2-week hypoxia treatment increases the sociability in adolescent mice without locomotor activity change and anxious influence.

Besides the alterations in social behavior, we sought to examine the impact of hypoxia treatment on working memory, another sensitive function in the adolescent stage. To investigate hypoxia’s influence on working memory, Y-maze test was performed after the three-chamber social approach test. Mice have an innate curiosity to visit the arm they have not entered. Therefore, the effective working memory can be reflected by spontaneous alteration in Y-maze. Mice treated with hypoxia showed higher spontaneous alteration than the control mice, *p* = 0.0471 (Figure 1C). The result indicates that hypoxia might enhance mice reference working memory at adolescent age.

Having found the impact of hypoxia on reference working memory, we further investigated hypoxia’s influence on spatial learning and memory through the Morris Water Maze experiment. Day 1 visible platform test was performed to exclude motor or visual obstacles, and no significant differences in travel latency, *p* = 0.1643 (Figure 1D), or distance, *p* = 0.5245 (Figure 1E) were detected between control and hypoxia-treated mice. After day 2–5 training, the control mice boarded the platform more quickly with shorter distances traveled. However, hypoxia-treated mice traveled longer latency, *p* = 0.7086 (Figure 1F, Day 5, control versus hypoxia), and distance, *p* = 0.6121 (Figure 1G, Day 5, control versus hypoxia), to find the hidden platform than the control ones, though not significant. On day 6 probe test, the control mice showed a non-significant trend toward passing the platform a greater number of times than the hypoxia-treated mice, *p* = 0.0580 (Figure 1H). Additionally, hypoxia-treated mice spent comparable time in the platform-located SW quadrant, *p* = 0.5679, (Figure 1I) and similar latency for first crossing the platform with the control mice, *p* = 0.3080 (Figure 1J). These results indicate that 2-week hypoxia treatment has no obvious influence on the spatial learning and memory in adolescent mice. In summary, intermittent hypoxia treatment enhanced sociability and facilitated working memory while barely affecting spatial learning and memory in adolescent mice.

To detect whether sex plays a role in our experiment, the behavioral outcomes were analyzed in a sex-dependent manner. Both male and female mice showed a similar enhancement in social exploring time in stage I, *p* < 0.001. For the social index comparison, however, only female mice with hypoxia exposure showed a significant increase compared with the female control mice (control, 0.04741 ± 0.06256; hypoxia, 0.2663 ± 0.05260), *p* = 0.0153, indicating an elevation in sociability, but not in male (control, 0.2149 ± 0.05749; hypoxia, 0.3149 ± 0.05625), *p* = 0.2311 (Supplementary Figure 2A). In stage II, despite the prolonged trend in interaction time with stranger 2 shown in hypoxia-treated female mice, *p* = 0.0835, no significant difference was observed between the two treatment groups. In both sexes, the social index did not reach the statistical difference. Male (control, 0.3268 ± 0.07044; hypoxia, 0.2196 ± 0.08055), *p* = 0.3351; female (control, 0.1846 ± 0.06572; hypoxia, 0.2953 ± 0.06723), *p* = 0.2542 (Supplementary Figure 2B). In the Y-maze test, hypoxia-treated male mice performed better in spontaneous alteration, *p* = 0.0431. Two groups of female mice showed comparable spontaneous alteration, *p* = 0.4989 (Supplementary Figure 2C). Furthermore, female mice’s spatial learning and memory were found more vulnerable to hypoxia exposure in the Morris Water Maze. Between two groups, there was no difference in latency. Male, *p* = 0.6091, female, *p* = 0.1271 (Supplementary Figure 2D). So as well in distance arriving the platform in Day 1. Male, *p* = 0.7337, female, *p* = 0.4704 (Supplementary Figure 2E). Comparing two treatment groups, there was no difference after 4-day training. In latency, male, *p* = 0.9994; female, *p* = 0.1956 (Day 5, control versus hypoxia, Supplementary Figure 2F). In distance, male, *p* = 0.9998; female, *p* = 0.1961 (Day 5, control versus hypoxia, Supplementary Figure 2G). Female control mice crossed the platform more times than those in the hypoxia group in a non-significant trend, *p* = 0.0587. Male, *p* = 0.5690 (Supplementary Figure 2H). Mice treated with hypoxia took about the similar residence time in the platform quadrant. Male, *p* = 0.9171; female, *p* = 0.3369 (Supplementary Figure 2I). Similar time was spent reaching the platform. Male, *p* = 0.8031; female, *p* = 0.2601 (Supplementary Figure 2J). Those results imply hypoxia increases sociability in female mice, enhances spatial working memory more obviously in male mice, and might affect spatial learning and memory in female mice.

### Hypoxia upregulates CNTNAP2 expression in the mouse brain

**FIGURE 2:**
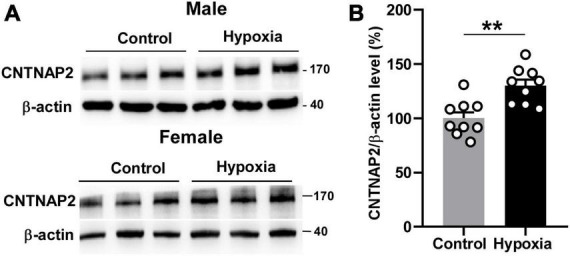
Hypoxia upregulated CNTNAP2 expression. **(A)** Hypoxia treatment increased CNTNAP2 expression level in the hippocampus of male and female mice. **(B)** Quantification of Western Blot results. The ratio of CNTNAP2 to corresponding β -actin protein level was normalized to the average of the control group. The values represent means ± SEM (*n* = 6 male **+** 3 female in each group), **p < 0.01 by unpaired *t*-test.

As mentioned in the introduction, hypoxia tends to affect neurodevelopment in younger ages. Since hypoxia treatment significantly enhanced sociability and working memory in adolescent mice, we hypothesized that the underlying mechanism might be associated with ASD-related molecules. ASD patients have reduced CNTNAP2 expression level (Sampath et al., 2013; Maekawa et al., 2015), while *Cntnap2* knockout mice and rats display pronounced impairments in sociability (Penagarikano et al., 2011; Scott et al., 2020). *Cntnap2* knockout mice also have working memory deficits (Rendall et al., 2016). These findings underscore the essential role of CNTNAP2 in sociability and working memory. Hence, we further investigated whether the enhanced sociability and working memory by hypoxia is related to CNTNAP2 expression levels, using 2-month mice brain tissue under hypoxia. In the mouse hippocampus, CNTNAP2 protein expression level was increased from 100 ± 5.45% to 130.0 ± 5.59% by hypoxia treatment, *p* = 0.0014 (Figures 2A, B). The similar increase in CNTNAP2 expression was observed in both male and female mice, 121.1 ± 4.61% vs. 100 ± 4.59% in male mice, *p* = 0.0089 (Supplementary Figure 3A), and 147.9 ± 5.36% vs. 100 ± 15.83% in female mice, *p* = 0.0457 (Supplementary Figure 3B). These results demonstrate that hypoxia treatment increased CNTNAP2 expression.

### Human *CNTNAP2* gene promoter cloning and transcription start site mapping

It has been well established that hypoxia upregulates target gene expression through the transcriptional regulation of HIF-1α. Although *CNTNAP2* is one of the largest genes in the human genome (Nakabayashi and Scherer, 2001), its transcriptional regulation remains largely unknown. To study the potential regulatory relationship between hypoxia and CNTNAP2, we systematically investigated the transcriptional regulation of the human *CNTNAP2* gene.

**FIGURE 3:**
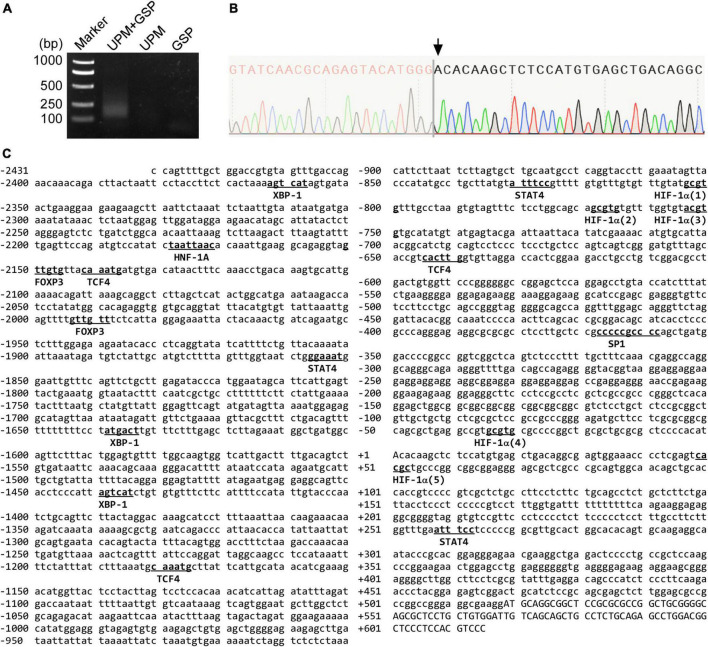
The sequence map of the human *CNTNAP2* gene promoter. **(A)** 5′-RACE assay was conducted to map the CNTNAP2 transcription start site (TSS). The PCR product was analyzed on a 2% agarose gel. USP = universal primer, GSP = CNTNAP2 gene specific primer. **(B)** The 5′-RACE PCR product was cloned into the pRACE vector and sequenced to identify the TSS. The TSS is the first nucleotide after the SMARTer oligonucleotide, as indicated by the arrow. **(C)** The sequence of the human CNTNAP2 gene promoter. A 2,868 bp 5′-flanking region of the human CNTNAP2 gene was amplified from HEK genomic DNA. The TSS Adenine is designated +1. The putative binding sites for computationally predicted transcription factors are underlined.

First, we conducted 5′-rapid amplification of cDNA ends (RACE) assay to identify the transcription start site (TSS) of the human *CNTNAP2* gene. Since CNTNAP2 is specifically expressed in the nervous system, total RNA was extracted from the human fetus brain sample to serve as the 5′-RACE assay template. PCR reactions from three independent experiments generated a ∼130 bp band (Figure 3A). The sequencing results identified the TSS at 518 bp upstream of the translation start site ATG, which began with adenine and was designated +1 (Figure 3B). Putative human transcription factors binding to the 2,868 bp 5′-flanking region of the human *CNTNAP2* gene were predicted by the PROMO online tool (Messeguer et al., 2002) with a dissimilar margin of up to 1%. The computational searching results revealed that the human *CNTNAP2* promoter contains putative *cis-acting* elements for several transcription factors, including X-box binding protein 1 (XBP-1), hepatocyte nuclear factor 1 homeobox A (HNF-1A), forkhead box protein P3 (FOXP3), signal transducer and activator of transcription 4 (STAT4), transcription factor 4 (TCF4), HIF-1α, and specificity protein 1 (SP1) (Figure 3C).

### Functional analysis of the human *CNTNAP2* gene promoter

**FIGURE 4:**
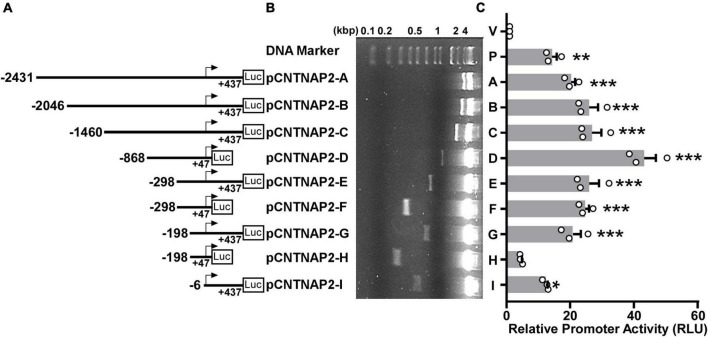
Deletion analysis of the human *CNTNAP2* gene promoter. **(A)** Schematic diagram of the human *CNTNAP2* promoter deletion constructs. Various lengths of the human CNTNAP2 gene 5′ flanking region were cloned into the pGL3-Basic promoter upstream of the Firefly luciferase gene (Luc). The numbers represent each fragment’s start and end positions relative to the transcription start site (TSS). **(B)** The plasmids used in the luciferase reporter assay were confirmed by restriction enzyme digestion, and the digested products were analyzed on a 1.5% agarose gel. **(C)** Functional analysis of human *CNTNAP*2 gene promoter. pCNTNAP2 plasmids were co-transfected with pCMV-Luc into PC12 cells. 24 h post-transfection, cells were harvested for the dual-luciferase reporter assay. The relative luciferase activities were calculated by normalizing Firefly luciferase activities to the Renilla luciferase activities and represented by relative luciferase units (RLU). The activity of the pGL3-Basic vector (marked as V) was designated as 1 RLU, and the pGL3-Promoter (marked as P) served as the positive control. The values represent means ± SEM (*n* = 3), *p < 0.05, **p < 0.01, ***p < 0.001 by Dunnett’s multiple comparisons test, compared with the pGL3-Basic control.

To analyze the function of the human *CNTNAP2* gene promoter, we cloned a 2,868 bp 5′-flanking region of the human *CNTNAP2* gene from HEK genomic DNA. Deletion fragments of the 2,868 bp 5′-flanking region were cloned into the pGL3-Basic vector (Figure 4A). The pGL3-Basic vector contains a Firefly luciferase reporter gene but lacks a eukaryotic promoter and enhancers. Inserting a functional promoter upstream of the Firefly luciferase gene can drive the luciferase expression. The relative promoter activity is reflected by the relative luciferase units (RLU) levels. *CNTNAP2* promoter fragments inserted into the vector were confirmed by Sanger sequencing and enzyme digestion (Figure 4B). To examine the transcriptional activation, *CNTNAP2* promoter fragments pCNTNAP2-A to I were transfected into PC12 cells, followed by luciferase reporter assay. pGL3-Basic (V) and pGL3-Promoter (P) were transfected simultaneously as negative and positive controls.

Compared with the empty vector pGL3-Basic (1 RLU), pGL3-Promoter showed robust promoter activity of 14.3 ± 1.49 RLU (*p* = 0.0025). The longest construct 2,868 bp pCNTNAP2-A (−2,431 to +437 bp) also showed significant luciferase activity (20.25 ± 1.33 RLU, *p* < 0.001, Figure 4C) compared with pGL3-Basic, indicating that this 2,868 bp fragment contains the functional promoter region of the human *CNTNAP2* gene. A deletion of 385 bp from the 5′ end to generate pCNTNAP2-B (−2,046 to +437 bp) slightly elevated the promoter activity (25.89 ± 2.88 RLU, *p* < 0.001). A further deletion from −2046 bp to −1460 bp (pCNTNAP2-C) showed little effect on promoter activity (26.8 ± 2.99 RLU, *p* < 0.001). Deletions from both ends to generate pCNTNAP2-D (−868to +47 bp) gave rise to the highest promoter activity of 43.25 ± 3.65 RLU (*p* < 0.001), supporting that the TSS is located within the +47 bp 3′ boundary. To narrow the region that contains essential transcription machinery, three pairs of 5′ deletions with + 47 bp and + 437 bp 3′ ends were generated. pCNTNAP2-E (−298 to +437 bp), pCNTNAP2-G (−198 to + 437 bp), and pCNTNAP2-I (−6 to +437 bp) with +437 bp 3′ end showed dwindling but significant promoter activities (E: 25.9 ± 3.18 RLU, *p* < 0.001; G: 20.85 ± 2.50 RLU, *p* < 0.001; I: 12.37 ± 0.54 RLU, *p* = 0.0105), indicating that the essential transcription machinery is within the −6 bp 5′ boundary. While pCNTNAP2-F (−298 to +47 bp) displayed decent promoter activity (24.62 ± 1.31 RLU, *p* < 0.001), further deletions at the 5′ end to pCNTNAP2-H (−198 to +47 bp) substantially diminished promoter activity to 4.54 ± 0.28 RLU (*p* = 0.8494), indicating some essential elements between −298 and −198 bp.

Taken together, these results indicate that the TSS is between −6 to +47 bp, supporting the TSS mapped in Figure 3B; the 345 bp fragment pCNTNAP2-F (−298 to +47 bp) has the minimum promoter activity required for transcription; and the promoter region −298 to −198 bp may contain inhibitory *cis-acting* elements.

### Hypoxia upregulates *CNTNAP2* promoter activity via functional HREs

By analyzing the sequence, we identified five putative HIF-1α binding sites containing 5′-A/(G)CGTG hypoxia-responsive elements (HRE) in the human *CNTNAP2* promoter (Figure 3C): HRE1 (5′ gcgtg, −804 to −800 bp), HRE2 (5′ gcgtg, −769 to −765 bp), HRE3 (5′ acgtg, −754 to −750 bp), HRE4 (5′ gcgtg, −35 to −31 bp), and HRE5 (reverse strand 5′ gcgtg, +49 to +53 bp).

**FIGURE 5:**
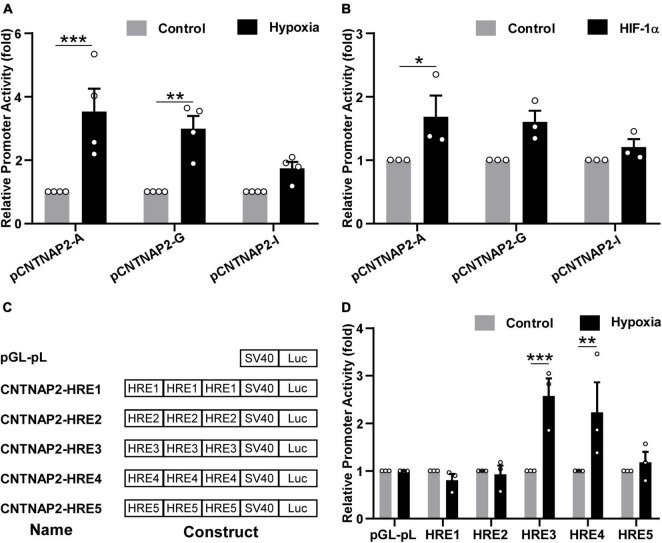
Hypoxia upregulated CNTNAP2 promoter activity via functional HREs. **(A)** Hypoxia upregulated CNTNAP2 promoter activity. pCNTNAP2-A containing HRE1-5, pCNTNAP2-G containing HRE4-5, and pCNTNAP2-I without HREs were co-transfected with pCMV-Luc into HEK cells followed by hypoxia treatment (2% O_2_) for 24 h. Hypoxia increased the promoter activities of pCNTNAP2-A and pCNTNAP2-G while showing little effect on pCNTNAP2-I (*n* = 4, two-way ANOVA, treatment is considered as an independent variable, *F* (1, 18) = 38.05, *p* < 0.0001). **(B)** HIF-1α upregulated CNTNAP2 promoter activity. pCNTNAP2-A, G, and I were co-transfected with HIF-1α or vector and pCMV-Luc into HEK cells. Twenty-four hours post-transfection, cells were harvested for the dual-luciferase reporter assay. HIF-1α enhanced the promoter activities of pCNTNAP2-A and pCNTNAP2-G but did not affect pCNTNAP2-I activity [*n* = 3, two-way ANOVA, treatment effect, *F* (1, 12) = 13.98, *p* = 0.0028]. **(C)** Schematic diagram of CNTNAP2-HRE constructs. The name and construct of each plasmid were as illustrated. Triple HRE1-5 was cloned into the pGL-pL vector without any cis-acting enhancers. **(D)** Screening of functional HREs responsive to hypoxia treatment. HRE constructs and pGL-pL vector were co-transfected with pCMV-Luc into HEK cells and treated under hypoxia (2% O_2_) for 24 h. HRE3 and HRE4 were activated by hypoxia treatment, while HRE1, 2, and 5 were not reactive to the hypoxia condition [*n* = 3, two-way ANOVA, treatment effect, *F* (1, 24) = 11.7, *p* = 0.0022]. The values represent means ± SEM, *p < 0.05, **p < 0.01, ***p < 0.001 by Sidak’s multiple comparisons test, compared with the control column of each plasmid.

To investigate whether the human *CNTNAP2* promoter can be regulated by hypoxia condition, pCNTNAP2-A (−2,431 to +437 bp) containing HRE1-5, pCNTNAP2-G (−198 to +437 bp) containing HRE4-5, and pCNTNAP2-I (−6 to +437 bp) with no HREs were transfected into HEK cells and treated with hypoxia condition (2% O_2_) for 24 h (Figure 5A). The promoter activities of pCNTNAP2-A and pCNTNAP2-G were significantly increased by hypoxia treatment to 3.53 ± 0.72 (*p* < 0.001), and 2.98 ± 0.40 (*p* = 0.0023) folds, respectively, while the activity of pCNTNAP2-I without HREs was not affected (1.74 ± 0.20 folds, *p* = 0.387). Similarly, co-transfected HIF-1α elevated the activities of pCNTNAP2-A (1.68 ± 0.33 folds, *p* = 0.0344) and pCNTNAP2-G (1.60 ± 0.18 folds, *p* = 0.0659) but not pCNTNAP2-I (1.20 ± 0.13 folds, *p* = 0.7772, Figure 5B), indicating that *CNTNAP2* promoter contains functions HREs.

To further explore the functional HREs in the *CNTNAP2* promoter, triple HRE1-5 were cloned into the pGL-pL vector and treated with hypoxia (Figure 5C). The activities of HRE3 and HRE4 were significantly increased by hypoxia to 2.57 ± 0.37 folds (*p* < 0.001) and 2.23 ± 0.63 folds (*p* = 0.0051) respectively. In contrast, activities of HRE1 (0.81 ± 0.13 fold, *p* = 0.992), HRE2 (0.93 ± 0.18 fold, *p* > 0.9999), and HRE5 (1.18 ± 0.22 folds, *p* = 0.9947) were not affected, suggesting that HRE3 (5′ acgtg, −754 to −750 bp) and HRE4 (5′ gcgtg, −35 to −31 bp) in the human *CNTNAP2* promoter are functional (Figure 5D). In summary, these results demonstrate that hypoxia upregulates CNTNAP2 expression by interacting with functional HREs in the CNTNAP2 promoter.

## Discussion

In the current study, we found that intermittent hypoxia treatment enhanced social behaviors and working memory in adolescent mice, potentially through upregulating CNTNAP2 expression. Hypoxia treatment increased CNTNAP2 protein expression in mouse brains and enhanced *CNTNAP2* promoter activity. *CNTNAP2* promoter contains two functional HREs that can be activated by hypoxia treatment.

Adolescence is the second critical stage for brain development and behavior development. Cortical thinning and white matter maturation occur in adolescence, leading to working memory maturation (Ostby et al., 2011). Fuhrmann et al. (2015) proposed a model of plasticity in which plasticity reaches its peak during adolescence, dedicated to social behavior modifications. During this sensitive period, social behaviors can be affected by environmental factors like stress. Adolescents are susceptible to social and physical stress. Adolescents with siblings addicted to alcohol and drugs tend to have imitative behavior (Thomas et al., 2022). Adolescent rats suffering from induced physical and psychological stress exhibited spatial memory impairment, reduced anxiety, and increased reactive oxygen species (ROS) levels (Mousavi et al., 2019). Since hypoxia is reported as an environmental risk factor inducing oxidative stress (McGinnis et al., 2014), it might also affect adolescent development. Our results demonstrate that sociability and working memory are enhanced by intermittent hypoxia treatment in adolescence, supporting the plasticity during this critical development stage. This finding broadens the current knowledge of hypoxia’s impact on neurodevelopment from prenatal and perinatal stages to adolescence.

The protective effect of intermittent hypoxia treatment in adolescence should not be surprising, as previously reported in animal and human studies of other developmental stages. For example, intermittent hypoxia training before behavior deterioration prevented memory deficits in the 3xTg-AD mouse model by increasing EPO and brain-derived neurotrophic factor (BNDF) (Ryou et al., 2021). Notably, the intermittent hypoxia treatment on healthy adolescent mice in our study can relate to the altitude exercises gradually springing up in the training of athletes. Studies reported that the age of athletes involved is around teens to twenties in adolescence. Under hypoxia training, muscle oxidative capacity is promoted (Geiser et al., 2001). A cohort study reported that athletes training at high altitudes achieved better performances (Sharma et al., 2018). Furthermore, a randomized controlled trial conducted on obese adolescents showed that hypoxia training united with normoxia helps reshape morphology and ameliorate exercise performance (Britto et al., 2020). Normoxic glucose tolerance is also improved after hypoxia training (De Groote et al., 2018). Brief, intermittent hypoxia exposure also showed clinical therapeutic effects on several neurological diseases by inhibiting proinflammatory mediators production and enhancing innate immune function (Navarrete-Opazo and Mitchell, 2014). The above studies imply hypoxia’s protective effect on adolescence. However, the effect on sociability has not been explored. Our study is the first to report the role of hypoxia in sociability and memory in healthy adolescent mice.

However, the effect of hypoxia treatment can be influenced by many variable factors, such as the oxygen level, the frequency and length of treatment, and the age when receiving treatment. In our study, social behaviors and short-term memory (working memory) was significantly enhanced, while long-term memory (spatial learning and memory) was not affected. The intermittent hypoxia treatment in our study may have mixed effects on different phases of memory consolidation. As reported by Pearson et al. (2010), C57BL/6J mice show no obvious preference in three-chamber approach test, which aligns with the no remarkable change in social novelty preference in our stage II. Our findings of hypoxia’s protective effect may contradict its previously reported adverse impacts in prenatal and perinatal stages. For example, Vázquez-Borsetti et al. (2016) reported that a 20-min hypoxia-ischemia insult on perinatal rats led to social deficits and loss of neurons. However, hypoxia studies on prenatal and perinatal rodents often apply artery ligation to induce hypoxia-ischemia exposure (Wang et al., 2021a), which is much harsher than the hypoxia treatment in our study. Driscoll et al. (2018) showed that 10% oxygen applied on pregnant Sprague-Dawley rats transiently for 3, 5, or 7 min combined with ligation of the infra-renal abdominal aorta and uterine arteries caused social deficits and inflammation in postcesarean pups at postnatal day 30. Although their hypoxia treatment (10% oxygen, 3, 5, or 7 min) seems milder than our hypoxia condition, the ischemia induced by artery ligation and the following reperfusion injury could play a significant role in the offspring’s phenotype. Moreover, since the fetus and newborns are frail, experiments at these stages can emphasize the detrimental side of hypoxia. Our study provides insights into adolescents, in which stage the mice may have a higher adaptability to environmental alterations.

The sex difference was noticed in our experiment (Supplementary Figure 2). Both male and female mice showed enhanced sociability in stage I, while hypoxia-treated female mice had significantly increased social index. In the Y-maze test, hypoxia-treated male mice performed better in visiting different arms in a row with the higher spontaneous alteration. Furthermore, results from the Morris Water Maze showed that female mice’s spatial learning and memory were more vulnerable to hypoxia exposure. Of note, an article mentions that hypoxia decreased the capacity of Complex II respiratory in male fetuses only, indicating a sex-dependent effect (Smith et al., 2022). Snyder et al. reported that androgens modulate chronic intermittent hypoxia’s effects on the brain and behavior (Snyder et al., 2018), which may underlie the sex difference in sociability and memory observed in our study.

To further investigate the potential mechanisms underlying hypoxia’s effect on mouse behaviors, we analyzed the CNTNAP2 expression level in the mouse brain sample. Hippocampus has been implicated in the social and memory impairment in ASD (Banker et al., 2021). Combining the enhanced sociability and compound memory alterations described above, we examined the hippocampal CNTNAP2 expression level. CNTNAP2 protein expression was increased in the mouse brain after hypoxia treatment. Although CNTNAP2 is not the only target upregulated by hypoxia, our results suggest that CNTNAP2 may be the critical molecule regulating social behaviors and working memory. CNTNAP2 loss function or malfunction caused by pathogenic mutations has been implicated in ASD pathogenesis in human studies (Bakkaloglu et al., 2008; Penagarikano and Geschwind, 2012). In animal studies, *Cntnap2* knockout mice and rats consistently display characteristic ASD phenotypes (Penagarikano et al., 2011; Scott et al., 2020). *Cntnap2*-KO mice also show profound deficits in working and reference memory (Rendall et al., 2016). In the current study, we found that hypoxia treatment increased CNTNAP2 expression in the mouse brain and enhanced social behaviors and working memory. Our results, together with previous findings, could indicate a dose-dependent effect of CNTNAP2 on social behaviors and working memory. However, more concrete evidence, such as comparisons among knockout, hemizygous, homozygous, and transgenic mice, are warranted to draw decisive conclusions. CNTNAP2 is also related to prefrontal cortex (PFC), a key brain region regulating social behavior. mPFC social representative signal was attenuated in CNTNAP2^–/–^ mice (Levy et al., 2019). Further studies could investigate hypoxia-induced CNTNAP2 alteration in neural circuits and PFC. In addition to CNTNAP2, the effects of genes involved in hypoxia-associated signaling pathway on sociability and working memory should be investigated.

Although abnormal expressions of CNTNAP2 have been indicated in ASD, the transcriptional regulation of the human *CNTNAP2* gene remains elusive. Thus far, no study has systematically analyzed the transcriptional regulation of the human *CNTNAP2* gene. Only three transcription factors were reported to regulate *CNTNAP2* gene expression: Forkhead box protein P2 (FOXP2) (Vernes et al., 2008), Transcription factor 4 (TCF4) (Forrest et al., 2012), and Storkhead box 1 (STOX1) (van Abel et al., 2012). Notably, CNTNAP2 expression is decreased in the hippocampus of Alzheimer’s disease (AD) patients due to the downregulation by STOX1 (van Abel et al., 2012), supporting the association between CNTNAP2 level and memory. We identified five HREs in the 2,868 bp human *CNTNAP2* gene promoter. However, only HRE3 (5′ acgtg, −754 to −750 bp) and HRE4 (5′ gcgtg, −35 to −31 bp) are functional HREs responsive to hypoxia treatment (Figure 5D). This conclusion is consistent with the results that the activities of pCNTNAP2-A containing HRE1-5 and pCNTNAP2-G containing HRE4-5 were upregulated by both hypoxia treatment and HIF-1α (Figures 5A, B). Our findings highlight the importance of experimental validation of putative transcriptional regulations since only two of the five HREs are functional.

The regulation of CNTNAP2 by hypoxia implicates some roles or consequences of CNTNAP2 in response to the oxygen homeostasis disturbance. Since the brain is especially susceptible to hypoxia (Sharp and Bernaudin, 2004), and CNTNAP2 is specifically expressed in the nervous system (Poliak et al., 1999), CNTNAP2 could be a key molecule of the brain in response to hypoxia conditions. Hypoxia is recognized as a risk factor at perinatal period (Aplin, 2000; Boman and De Butte, 2019), inducing ASD-like behaviors in offspring (Wang et al., 2021b). Besides injury in the cortex, chronic hypoxic brain damage also shows in synaptic loss in preterm infants (Wang et al., 2018) and synaptic plasticity deficits (Kazim et al., 2022). Synapse development is of much concern in brain development, and synaptic impairment has been observed in ASD patients and animal models. Many ASD-related genes contribute to synaptic alterations, including the hyperconnectivity of human neurons with SHANK2 mutations (Zaslavsky et al., 2019), the reduction in postsynaptic minimal excitatory current with PTCHD1-AS exon 3 destruction (Ross et al., 2020), and the facilitation in clustered synaptic genes transcription under POGZ variants (Markenscoff-Papadimitriou et al., 2021). Hence, hypoxia may promote ASD pathogenesis via synapse responses, especially when the synaptic proteins are coded by hypoxia-regulated genes. CNTNAP2 is a synaptic molecule with multiple roles in synaptic development and functions, such as axonal growth, dendritic arborization, synaptic transmission and plasticity (Anderson et al., 2012; Gdalyahu et al., 2015; Varea et al., 2015; Canali et al., 2018; Lazaro et al., 2019). The changes in CNTNAP2 levels under hypoxia conditions could shape the synaptic landscape dynamically in reaction to different oxygen statuses. Furthermore, CNTNAP2 and hypoxia are both implicated in ASD during early development. Hypoxia is a critical environmental risk factor for ASD pathogenesis at the perinatal stage (Aplin, 2000; Boman and De Butte, 2019), while born CNTNAP2 malfunction contributes to ASD pathogenesis in human and animal studies (Bakkaloglu et al., 2008; Penagarikano et al., 2011; Penagarikano and Geschwind, 2012; Scott et al., 2020). Our results demonstrate that CNTNAP2 is upregulated by hypoxia in adolescence. In this crucial period for brain development, hypoxia might play a protective role from ASD via upregulating CNTNAP2, a cell adhesion molecular and synaptic protein.

The regulation relationship will provide novel clues toward understanding the interaction between genetic and environmental factors in ASD pathogenesis. Future studies can further investigate the transcriptional regulation network under hypoxia, particularly the regulatory relationships with target genes involved in pathogenic conditions.

## Conclusion

In conclusion, our results showed for the first time that intermittent hypoxia treatment at the adolescent stage enhances social behaviors and reference working memory. Our study also reveals that hypoxia upregulates human *CNTNAP2* gene expression at the protein level. We identified the TSS and analyzed the transcriptional activation of the human *CNTNAP2* gene promoter. Hypoxia upregulates CNTNAP2 expression via two functional HREs in the *CNTNAP2* promoter. These results will provide novel insights into the impacts of hypoxia exposure on adolescent development and the potential interaction between CNTNAP2 and hypoxia in ASD pathogenesis.

## Data availability statement

The raw data supporting the conclusions of this article will be made available by the authors, without undue reservation.

## Ethics statement

The animal study was reviewed and approved by University of British Columbia and Wenzhou Medical University.

## Author contributions

WS conceived and designed the experiments. QZ, LX, YB, and PC performed the experiments. QZ, LX, FC, MX, YW, and WS analyzed and contributed reagents, materials, analytical tools. QZ, LX, YW, and WS wrote the manuscript. All authors reviewed the manuscript.
