# Supplementary material for: Intermittent hypoxia-induced enhancement of sociability and working memory associates with CNTNAP2 upregulation
Source: Front Mol Neurosci. 2023 Apr 6;16:1155047. doi: 10.3389/fnmol.2023.1155047 (PMC10118049; doi:10.3389/fnmol.2023.1155047)
Supplement: Supplementary file 1 [file Data_Sheet_1.pdf]

Supplementary Material

Intermittent hypoxia-induced enhancement of sociability and working memory associates with CNTNAP2 upregulation

Qing Zhang[1,2]†, Lu Xu[1]†, Yang Bai[1], Peiye Chen[1], Mengen Xing[1], Fang Cai[2], Yili Wu[1 *] and Weihong Song[1 *]

† These authors contributed equally to the work
* Corresponding author. Email: weihong@wmu.edu.cn, ORCID ID: 0000-0001-9928-889X; and wuyili@wmu.edu.cn

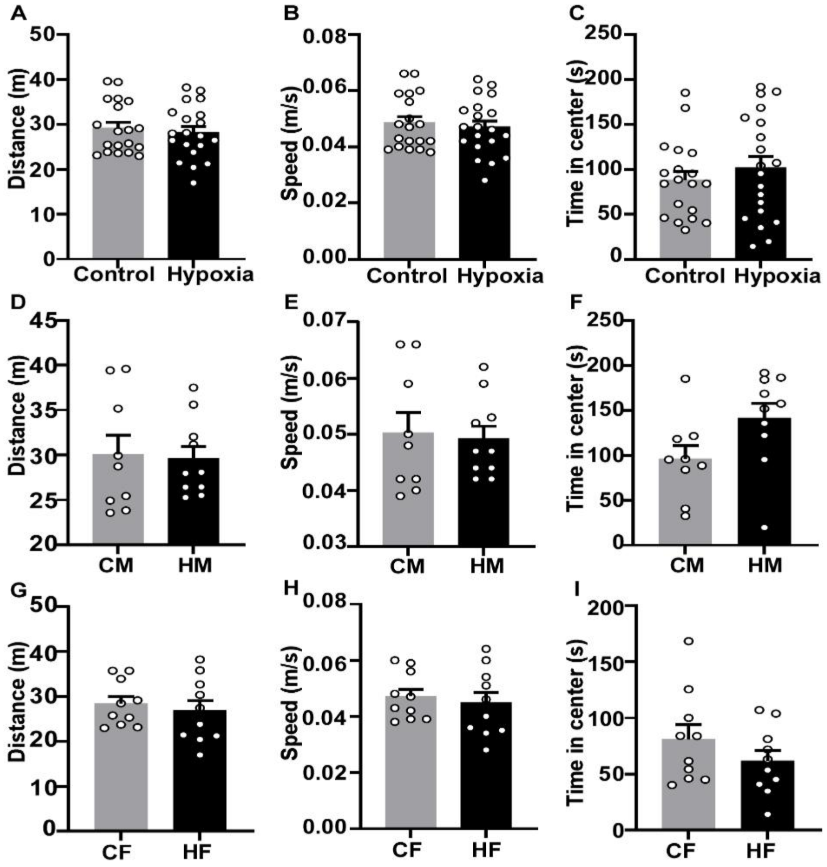

Supplement Figure 1

Supplementary Figure 1. Intermittent hypoxia treatment did not affect locomotor activity and anxiety of mice. Open field test was performed to investigate the locomotor activity and emotional alteration. (A-C) The outcomes were analyzed combing the male and female mice. Hypoxia exposure made no difference in distance, speed of mice and time in the center zone. (D-F) Data was analyzed in a sex-dependent manner. Male mice stimulated by hypoxia resembled the control mice in activity and emotional changes. (G-I) Similarly, female mice treated with hypoxia

had comparable performance with the control ones. CM = Control Male, HM = Hypoxia Male, CF = Control Female, HF = Hypoxia Female. The values represent means ± SEM. P values were calculated by unpaired t-test.

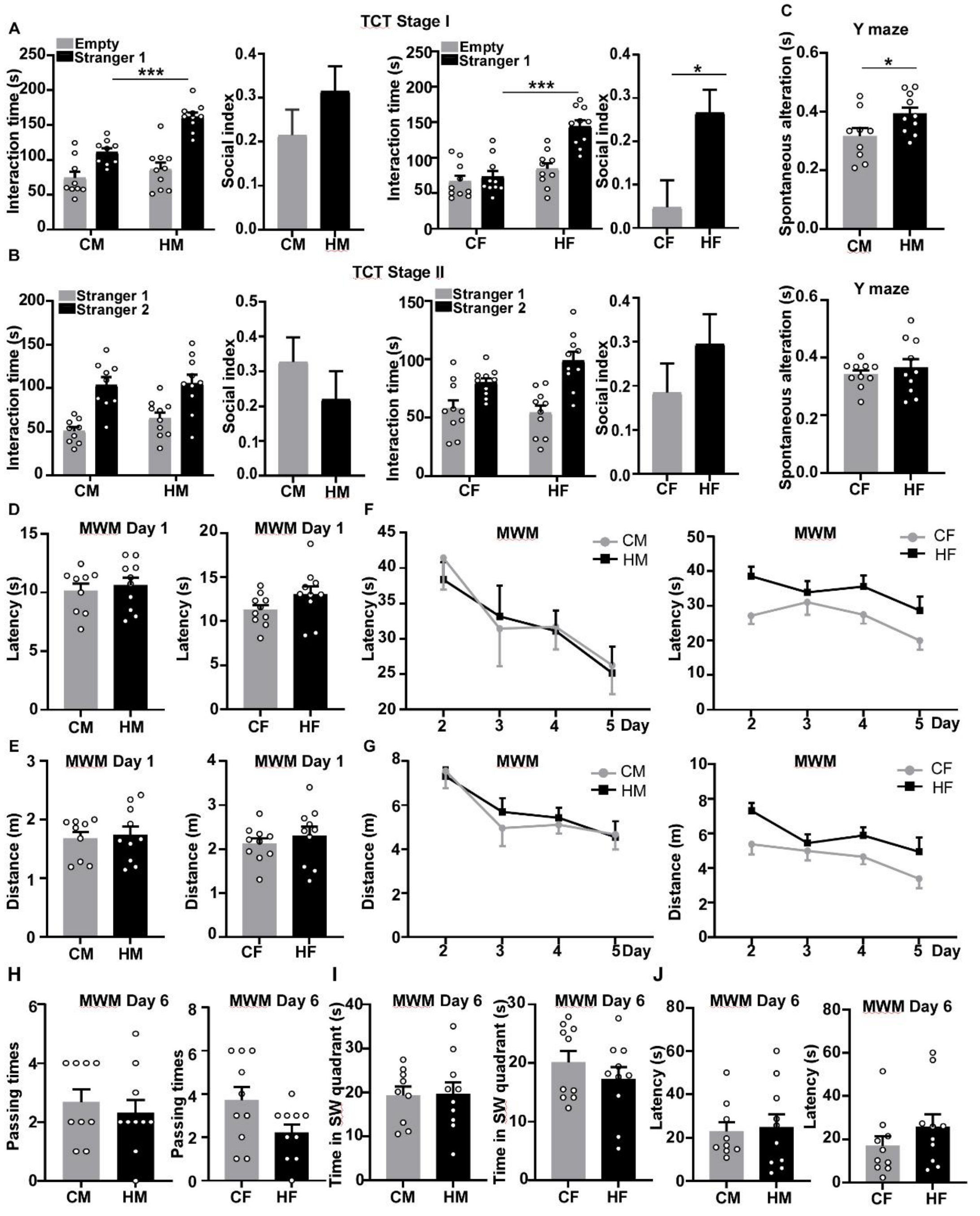

Supplementary Figure 2

Supplementary Figure 2. Intermittent hypoxia treatment affected behaviors in a sex-dependent manner. (A) Stage I sociability test. Two-way ANOVA was used, and the treatment is considered as an independent variable. Male, F (1, 34) = 16.25, p < 0.001, control, n =9, hypoxia, n = 10; female, F (1, 36) = 29.47, p < 0.001, control, n = 10; hypoxia, n = 10. The social index was the ratio of the difference to the sum of time interacting with stranger mouse 1 and empty cage (unpaired t-test). (B) Stage II social novelty preference test. Two-way ANOVA was applied, and the treatment

effect is an independent variable. Male, F (1, 34) = 1.054, p = 0.3118; female, F (1, 36) = 1.453, p = 0.2360. The social index meant the ratio of the difference to the sum of time interacting with stranger mouse 1 and stranger mouse 2 (unpaired t-test). (C) Y maze test. Spontaneous alteration represents the incidence of mice visiting different arms for three consecutive times. Unpaired t-test was used to calculate the p value. (D, E) Day 1 visible platform. There was no difference in the latency and distance boarding the platform. P values were calculated by unpaired t-test. (F-G) Day 2 to day 5 hidden platform training. Two-way ANOVA was used to detect the treatment effect in latency (male, F (1, 68) = 0.07656, p = 0.7829; female, F (1, 72) = 12.37, p < 0.001) and distance (male, F (1, 68) = 0.1468, p = 0.7028; female, F (1, 72) = 10.60, p = 0.0017). After training, mice in each group boarded the platform in a shorter time and a shorter distance. (H-I) Probe test on day 6. No significant differences were discovered. Unpaired t-test was used to calculate the p values. The values represent means ± SEM. *p < 0.05, **p<0.01, ***p < 0.001.

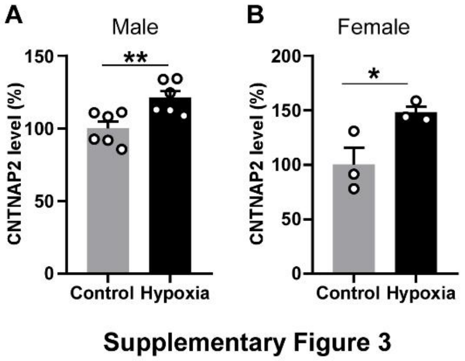

Supplementary Figure 3

Supplementary Figure 3. Intermittent hypoxia treatment elevated CNTNAP2 expression. We analyzed the CNTNAP2 protein expression level via sexual segregation. Quantification of Western Blot results in the hippocampus of male mice (A, n = 6) and female mice (B, n = 3). CNTNAP2 expression in the hippocampus was increased by hypoxia treatment. The values represent means ± SEM, *p < 0.05, **p < 0.01 by unpaired t-test.
